# Placental chorangiomas: Diagnostic challenges and contemporary approaches to fetal care

**DOI:** 10.1002/pmf2.70181

**Published:** 2025-12-04

**Authors:** Tiffany E. Deihl, May Abiad, Enaja Sambatur, Nikan Zargarzadeh, Ramen H. Chmait, Alireza A. Shamshirsaz, Stephen P. Emery

**Affiliations:** ^1^ Center for Innovative Fetal Intervention University of Pittsburgh School of Medicine UPMC Magee‐Womens Hospital Pittsburgh Pennsylvania USA; ^2^ Fetal Care and Surgery Center Boston Children's Hospital Harvard Medical School Boston Massachusetts USA; ^3^ Department of Obstetrics and Gynecology Division of Maternal‐Fetal Medicine Keck School of Medicine University of Southern California Los Angeles California USA

**Keywords:** chorioangioma, fetal anemia, fetal hydrops, placental chorangioma, placental tumor

## Abstract

Placental chorangiomas are the most common benign tumors of the placenta. Although most are small and asymptomatic lesions, giant chorangiomas are associated with an increased risk of adverse maternal and fetal outcomes. Therefore, in those affected by giant placental chorangioma, early diagnosis and intensive fetal monitoring are indicated to screen for fetal compromise and intervene as indicated. In some cases, expectant management or supportive therapies may serve a role. In the setting of fetal compromise, definitive treatment aimed at devascularization of the tumor has been described to gain pregnancy latency and avoid the risks of preterm delivery. Although multiple treatment options have been described, the optimal management of giant placental chorangioma remains undetermined. This narrative review serves to review the definition, diagnosis and pathophysiology of chorangioma, associated pregnancy complications, proposed management, and possible treatment options.

## 1 DEFINITION AND INCIDENCE

Placental chorangiomas (also known as chorioangioma) are benign tumors of the placenta that are derived from non‐trophoblast cells. The etiology of placental chorangioma is believed to reflect benign capillary endothelial proliferation within chorionic villi, possibly triggered by local hypoxia and abnormal angiogenesis during placental development [1, 2, 3]. Histologically, chorangiomas consist of small blood vessels that are embedded within the stroma of enlarged placental villi and are covered with a trophoblast layer [4]. While the definitive cause of the condition remains unknown, various risk factors have been associated with these tumors, including primiparity, maternal age greater than 30 years, multifetal gestation, maternal hypertension, maternal diabetes, and a female fetus [4, 5].

Incidental placental chorangiomas are found in approximately 1% of cases when the placenta is examined microscopically [4, 6]. However, clinically or ultrasonographically detected lesions are much less frequent, with reported incidences ranging from 0.1% to 0.16% [7, 8]. For instance, in a large retrospective study by Wou et al., only 23 chorangiomas were identified among 14,725 singleton placentas (0.16%) [9].

Giant chorangiomas, defined as tumors larger than 4 cm [6], are exceedingly rare, occurring in approximately 1 in 9000 to 1 in 50,000 pregnancies [10, 11]. Despite their rarity, these large lesions carry important clinical implications, as about half are associated with significant fetal and/or maternal complications requiring intervention or elective delivery [10, 12].

## 2 PROPOSED PATHOPHYSIOLOGY

Vascular channels exist within the chorangioma and act as arteriovenous shunts, which, in the setting of large and vascular tumors, can lead to fetal compromise. The chorangioma is the lowest resistance area in fetal circulation, leading to a preferential flow of blood from the fetus to the placental tumor [13]. In the case of a rapidly enlarging chorangioma, this “steal” phenomenon causes compromised perfusion of chorionic villi and leads to reduced delivery of essential nutrients and oxygen to the fetal circulation. Chronic shunting can be associated with uteroplacental insufficiency with subsequent chronic hypoxia, oligohydramnios, and growth restriction [14, 15]. In response to altered vascular dynamics caused by tumor shunting and the exposure to metabolic byproducts of the tumor, the fetal heart compensates to maintain tissue perfusion and is at risk for the development of cardiomegaly and heart failure [5, 16]. A compounding factor is the presence of severe fetal anemia that is thought to develop secondary to increased flow in the tumor leading to red cell sequestration as well as microangiopathic hemolysis [17]. Given the significant cardiac demands, cardiac dysfunction due to high‐output cardiac failure develops over time, ultimately leading to fetal hydrops and eventually fetal death [3].

## 3 DIAGNOSIS

The International Society of Ultrasound in Obstetrics and Gynecology (ISUOG) recommends that, during the routine anatomic survey, the location of the placenta, its relationship with the internal cervical os, and its appearance be described in order to identify abnormal findings, including hemorrhage, placental cysts, or placental masses [18]. A prenatal diagnosis of chorangioma is important given its potential association with adverse perinatal outcomes [1, 19, 20, 21]. Early diagnosis can greatly influence consequent fetal and maternal surveillance and allows for the potential of intervention.

**FIGURE 1:**
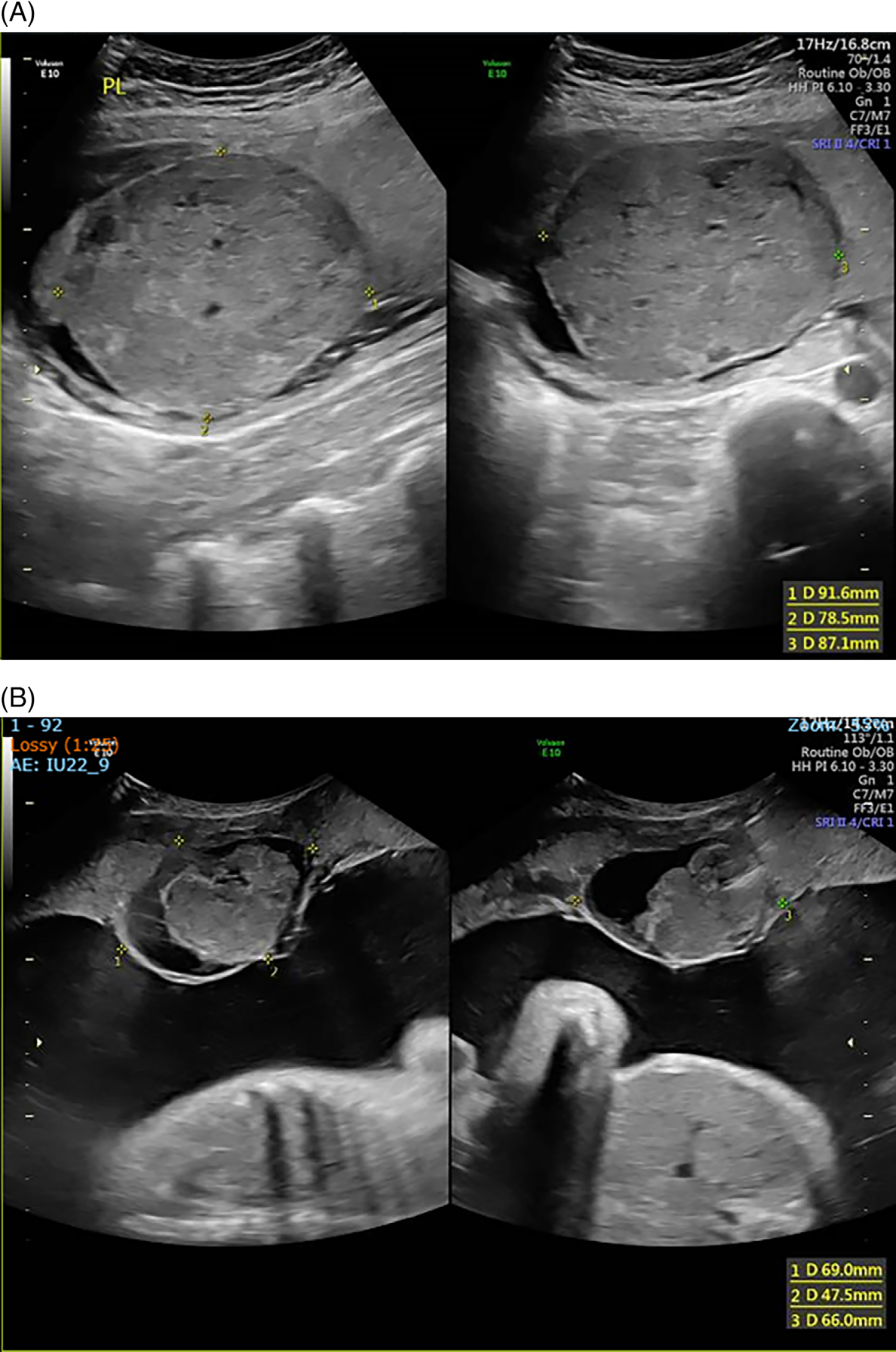
(A) Sonographic appearance of a giant placental chorangioma. Note the relatively homogeneous echotexture. (B) Giant chorangioma with a heterogeneous echotexture.

Ultrasound findings of chorangioma commonly include a hypo‐ or hyperechoic well‐circumscribed, rounded placental mass [22]. Of note, necrosis, degeneration, calcification, or hemorrhage within the tumor (Figure 1) may result in different ultrasonic echotexture [14]. They are commonly found in close proximity to the cord insertion site on the fetal surface of the placenta immediately beneath the chorionic plate, where they can protrude into the amniotic cavity [5, 14, 23, 24]. Application of color Doppler allows visualization of the feeding vessel(s) originating from the placental cord insertion and entering the placental mass, and of peri‐tumoral diffuse vascularization [3]. Color Doppler is instrumental in distinguishing chorangiomas from other placental lesions such as placental hematomas, chorangiocarcinoma, partial hydatidiform moles, teratomas, and degenerating fibroids [24, 25, 26, 27].

## 4 PREDICTING ADVERSE OUTCOMES

Chorangiomas are often minute and clinically unremarkable, usually recognized only as incidental findings during placental examination after birth. Giant placental chorangiomas (defined as > 4 cm), by contrast, are associated with an increased risk of adverse perinatal outcomes [20, 21]. In the most severe cases, signs of high‐output cardiac failure including cardiomegaly, polyhydramnios, increased velocity in the middle cerebral artery (MCA), and fetal hydrops are typically identified [1, 28, 29]. The size of the mass, presence of hydrops, and gestational age at the occurrence of cardiac failure have been reported to be the main determinants of perinatal outcome in pregnancies complicated by chorangioma [3, 7, 20].

The largest systematic review and meta‐analysis was performed by Buca et al. in 2020 and included 161 singleton pregnancies affected by placental chorangioma. In the 161 pregnancies managed expectantly, the perinatal mortality rate was 12.1%, comprising 8.2% intrauterine and 3.8% neonatal deaths [3]. Preterm delivery due to polyhydramnios occurred in 68% of cases, placing those infants at risk for morbidity related to prematurity [3]. Evidence suggests that the risk of adverse outcomes increases with both tumor size and the presence of fetal hydrops, the latter being associated with a particularly high perinatal mortality rate of 40.5% [3, 30].

Based on the concept of “steal phenomenon,” some researchers have hypothesized that the vascularity of the tumor may be an independent risk factor for fetal complications regardless of the tumor size. This hypothesis would suggest that color flow and power‐Doppler flow imaging may give valuable information on the prognostic evaluation of large chorangioma. However, because there is no standardized method to grade the vascular intensity of chorangiomas on ultrasound, this screening approach is unlikely to be useful in routine practice at present [14]. Three‐dimensional color Doppler acquisition and placental magnetic resonance imaging (MRI) have recently been reported as adjuncts to placental imaging, which can offer more information about the tumor and its vascular distribution [31, 32].

Some investigators have sought to assess tumor severity through indirect markers of chorangioma vascularity. For instance, Sepulveda et al. described three cases of large placental chorangiomas, noting that the two pregnancies with poor outcomes involved tumors located near the umbilical cord insertion with prominent branching vessels. In line with this, both Ma et al. and Liu et al. reported statistically significant differences in the cord–tumor distance when comparing pregnancies with adverse versus favorable outcomes [14, 28, 29].

## 5 POTENTIAL COMPLICATIONS

Maternal complications associated with placental chorangioma include symptomatic polyhydramnios, preterm premature rupture of membranes (PPROMs), preeclampsia, and mirror syndrome [14, 33]. Other complications that have been described include maternal microangiopathic hemolytic anemia and thrombocytopenia, consumptive coagulopathy, placental abruption, and hemoglobinuria [10, 11, 14, 34, 35, 36, 37, 38]. Polyhydramnios is the most common maternal complication, occurring in 18%–35% of cases of giant chorangiomas [25].

Principal fetal complications include anemia, thrombocytopenia, fetal growth restriction, congestive heart failure, cardiomyopathy, non‐immune hydrops, and fetal death [10, 11, 34, 35, 38, 39]. The variation in fetal complications likely reflect the differences in the underlying angioarchitecture of the chorangioma. Lesions composed predominantly of capillaries with increased vascular area are more often associated with adverse fetal outcomes due to mechanisms such as the “steal” phenomenon, substrate competition, and red blood cell sequestration [40]. These can manifest as placental insufficiency, fetal growth restriction, oligohydramnios, anemia, and thrombocytopenia. Tumors with large or multiple AV shunts likely lead to complications related to “shunt physiology,” including fetal cardiomegaly, polyhydramnios, high‐output cardiac failure, and fetal hydrops. The overall perinatal mortality is estimated at 30%–40% [21, 41]. These findings are further illustrated in Table 1, which summarizes the characteristics and perinatal outcomes reported across multiple cohort studies of large placental chorangiomas.

**TABLE 1:** Characteristics and outcomes of patients with large placental chorangiomas.

Study	Number of patients	Mean GA (weeks) at diagnosis	Maximum tumor diameter (cm)	Hydrops, *n*(%)	Cardiomegaly, *n*(%)	Mean GA (weeks) at delivery	IUT performed, *n*(%)	Perinatal survival, *n*(%)
Agarwal 2023	34	24.5	7.1	NR	NR	37.0	4 (11.8)	30 (88.2)
Lim 2015	10	24.1	7.9	3 (30.0)	5 (50.0)	34.3	—	8 (80.0)
Liu 2013	16	27.6	5.9	1 (7.1)	3 (21.4)	36.4	—	14 (87.5)
Ghourab 2001	6	24.0	7.3	1 (16.7)	NA	35.0	—	5 (83.3)
Jauniaux 2000	9	22.9	5.3	1 (11.1)	1 (11.1)	37.2	—	8 (88.9)
Ma 2023	44	28.7	15.0	9 (20.5)	4 (9.1)	30.3	—	35 (79.5)
Zanardini 2010	19	28.6	6.3	NR	6 (31.6)	37.1	1 (5.3)	18 (94.7)
Saeed 2023	11	NR	22.0	3 (27.3)	1 (9.1)	34.2	2 (18.2)	10 (90.9)
Sepulveda 2003	11	26.0	7.8	1 (9.1)	1 (9.1)	35.3	—	9 (81.8)
Wu 2016	26	29.0	11.3	1 (3.8%)	1 (3.8%)	36.0	—	25 (96.2)

Abbreviations: GA, gestational age; IUT, intrauterine transfusion; NR, not reported.

## 6 ANTENATAL MANAGEMENT

Ultrasound findings concerning chorangioma warrant a referral to a tertiary hospital for further assessment, diagnosis, and follow‐up. On gray‐scale ultrasound, chorangiomas typically appear as well‐circumscribed, round, hypo‐, or hyperechoic masses located on the fetal surface of the placenta, often demonstrating internal vascularity on color Doppler. MRI is not routinely indicated but may be useful in the evaluation of large, heterogeneous placental masses to differentiate chorangioma from other placental tumors [14].

**FIGURE 2:**
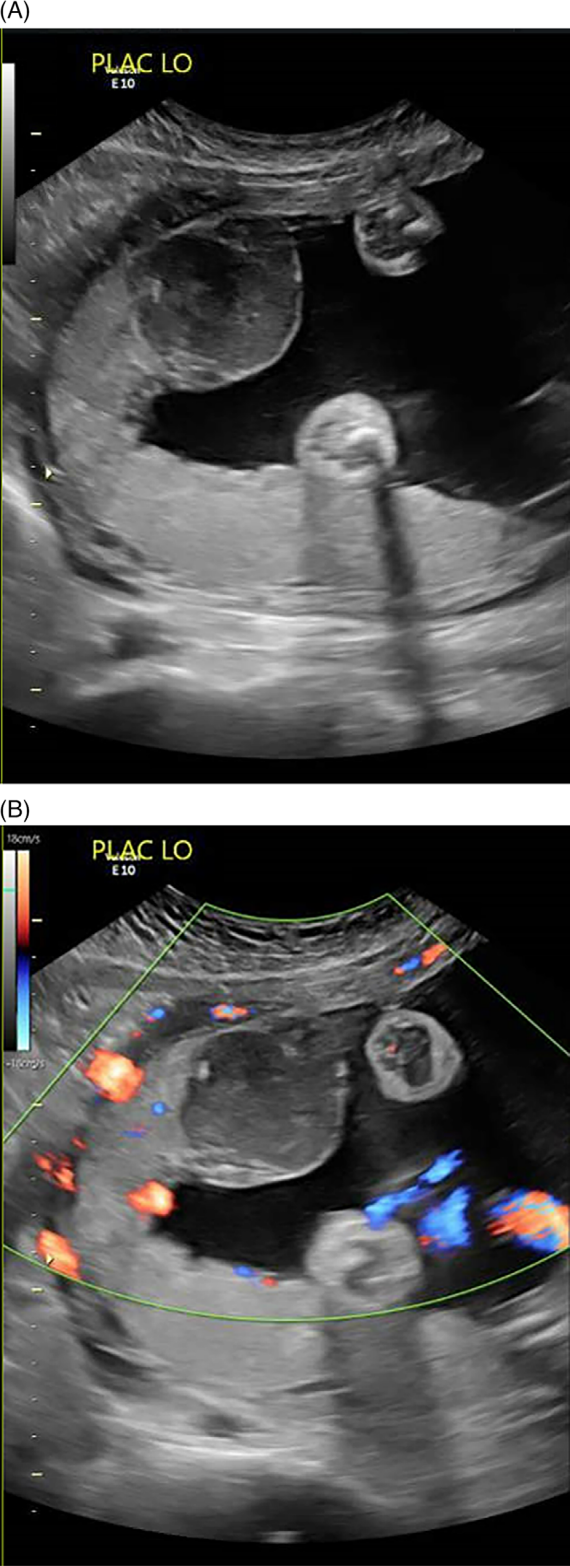
(A) Sonographic appearance of giant chorangioma with homogeneous echotexture. (B) Color Doppler interrogation demonstrates the absence of vascular flow within the tumor, consistent with spontaneous thrombosis.

**FIGURE 3:**
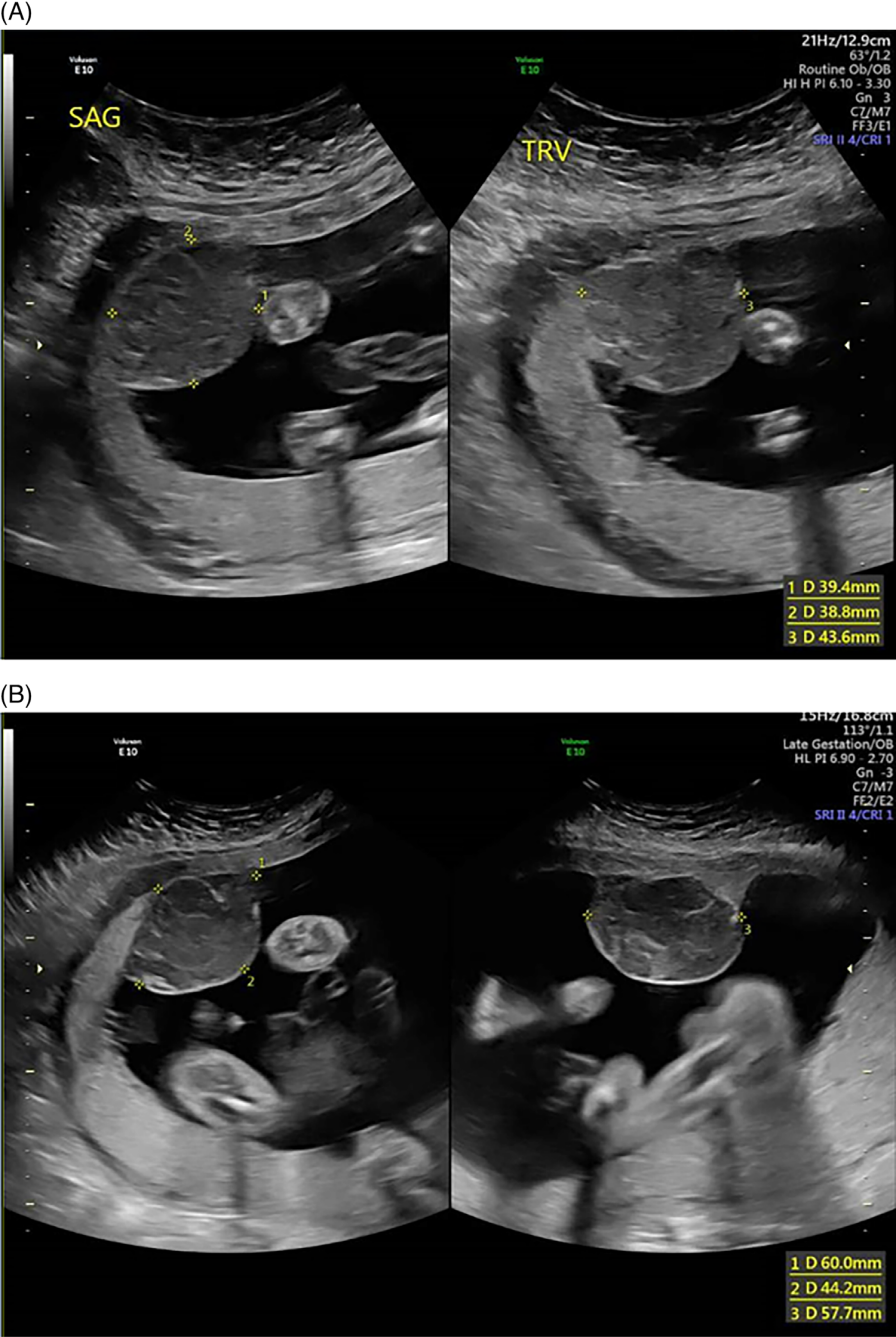
(A) Giant chorangioma at 20 weeks 3 days with a maximum diameter of 39 mm. (B) Same patient at 24 weeks 3 days now with a maximum tumor diameter of 60 mm.

Given the association between increasing tumor size and risk of adverse perinatal outcomes, pregnancies presenting with a large (≥4 cm) placental mass should undergo intensive fetal monitoring to screen for early signs of fetal compromise. Though spontaneous thrombosis and resolution of giant chorangiomas with subsequent favorable pregnancy outcomes have been reported, such events remain rare (Figure 2) [1, 28]. Complications related to chorangioma can present acutely and chorangiomas may grow rapidly (Figure 2). Although the frequency of follow‐up is not definitively established, serial fetal assessments every 1–2 weeks have been proposed as a reasonable option in these cases (Figure 3) [3].

In addition to monitoring the size of the chorangioma, assessment of the amniotic fluid index, assessment of the peak systolic velocity (PSV) within the MCA, and screening for the presence of fetal hydrops are recommended. Some authors have suggested monitoring for signs of abnormal cardiac function by measuring the fetal cardiothoracic circumference ratio (CC:TC ratio; > 0.50 consistent with severe cardiomegaly), high combined cardiac output (CCO > 400 mL/min/kg), and the presence of tricuspid regurgitation [16, 42]. However, given the expertise required to obtain these measurements and the lack of standardized interpretation, these cardiac parameters are not recommended as part of routine monitoring. Agarwal et al. recently outlined a stepwise management system for pregnancies complicated by chorangioma [42].

If complications develop late in pregnancy, planned delivery is a reasonable option. Unfortunately, most complications occur early in gestation, making iatrogenic preterm delivery inappropriate due to the high risk of neonatal morbidity and mortality secondary to prematurity. Furthermore, in cases complicated by hydrops, iatrogenic preterm delivery adds an additional burden to an already compromised fetus, thereby increasing the risk of perinatal death. In such circumstances, in utero therapy can be considered as an alternative approach to prevent fetal demise due to anemia or hemodynamic complications of the chorangioma and attempt to prolong the pregnancy [43]. Importantly, perinatal mortality remains high even in the setting of attempted therapies, and the scientific evidence for clinical treatment is limited to case reports and retrospective series.

## 7 POSSIBLE IN UTERO THERAPIES

### 7.1 Supportive therapies

Symptomatic polyhydramnios has been treated with therapeutic amnioreduction and/or transplacental pharmacotherapy with indomethacin (non‐selective cyclooxygenase [COX] inhibitor) or sulindac (COX‐2 inhibitor) with varying reported outcomes in the literature [12, 28, 34, 44, 45]. There is anecdotal evidence suggesting that amnioreduction may acutely worsen the fetus status, as it may reduce intra‐amniotic pressure, allowing increased “steal” of blood into the chorioangioma [46]. Transplacental digoxin has also been used to attempt to treat fetal cardiac dysfunction with varying outcomes [47]. Fetal anemia secondary to chorangioma has been managed with intrauterine blood transfusion, either as a stand‐alone therapy or as an adjunct prior to definitive treatment, with varying outcomes reported in the literature [48, 49, 50]. Of note, intrauterine transfusion for fetal anemia secondary to large chorioangiomas may require nearly twice the blood volume that is normally anticipated [51]. Although temporizing interventions such as these may have a role in management in some cases, the underlying pathophysiology is not resolved with these therapies and the perinatal mortality rate remains high [21].

### 7.2 Definitive treatment options

Definitive treatment refers to the goal of disrupting the tumor's blood supply, thereby arresting shunt physiology, “steal” phenomenon, red blood cell sequestration, and hemolysis. Multiple devascularization techniques have been reported in the literature, all with their own potential risks and benefits. Tables 2 and 3 present outcomes stratified by presence of hydrops and cardiomegaly, comparing expectant versus interventional management across multiple cohort studies.

**TABLE 2:** Characteristics and outcomes of patients with large placental chorangiomas, stratified by intervention and presence of fetal hydrops.

			Mean GA (weeks) at diagnosis	Mean GA (weeks) at intervention	Perinatal survival					
	Number of patients				Expectant management			Interventional management		
Study	Hydropic	Non‐hydropic			Overall	Hydropic	Non‐hydropic	Overall	Hydropic	Non‐hydropic
Agarwal 2023	3	6	NA	24.5	—	—	—	5/9	0/3	5/6
Lim 2015	3	7	24.1	24.0	4/5	—	4/5	4/5	2/3	2/2
Liu 2013	1	15	27.6	NR	14/16	1/1	13/15	—	—	—
Ghourab 2001	5	1	24.0	NR	5/6	0/1	5/5	—	—	—
Jauniaux 2000	1	8	22.9	32.0	—	—	8/8	0/1	0/1	—
Ma 2023	9^a^	35	28.7	NR	33/42	2/7	31/35	—	—	—

Abbreviations: GA, gestational age; NR, not reported.

^a^ Only 7 with explicit details on outcomes.

**TABLE 3:** Characteristics and outcomes of patients with large placental chorangiomas, stratified by intervention and presence of cardiomegaly.

				Perinatal survival					
		Mean GA (weeks) at diagnosis	Mean GA (weeks) at intervention	Expectant management			Interventional management		
Study	Number of patients			Overall	Cardiomegaly	No cardiomegaly	Overall	Cardiomegaly	No cardiomegaly
Agarwal 2023	34	24.5	24.5	24	—	—	5	5	0
Lim 2015	10	24.1	24.0	5	—	—	5	—	—
Liu 2013	16	27.6	NR	16	3	13	—	—	—
Ghourab 2021	6	24.0	NR	6	—	—	—	—	—
Jauniaux 2000	9	22.9	NR	9	1	8	—	—	—
Zanardini 2010	19	28.6	NR	10	—	10	8	6	2
Saeed 2023	11	NR	NR	0	1	—	2	2	0

Abbreviations: GA, gestational age; NR, not reported.

#### 7.2.1 Ultrasound‐guided percutaneous treatment

Embolization of the feeding vessel has been described using liquid agents—including ethanol, cyanoacrylate, and enbucrilate [28, 52, 53, 54, 55, 56]—as well as with microcoils [12, 57, 58, 59]. A major benefit of this technique is its minimally invasive nature—requiring only a 21‐gauge needle placed percutaneously under ultrasound guidance with maternal intravenous sedation and local anesthetic. Additionally, in the case of an anterior placenta, the entire procedure can be performed without entering the amniotic cavity. The disadvantage of using liquid agents for feeding vessel occlusion includes rapid hemodynamic changes within the tumor that may not be tolerated by the fetus, reflux of the liquid embolic material, and the potential passage of the embolic agents to the fetus through arteriovenous (AV) anastomoses within the chorangioma, as described by Voon et al. [56].

The use of microcoils, which cause vascular occlusion at the point of insertion, has been described in several publications with varying degrees of success [12, 57, 58, 59, 60]. Advantages of microcoil embolization include the ease and familiarity of the technique, as the placement is technically similar to obtaining vascular access for percutaneous umbilical blood sampling/intrauterine transfusion (PUBS/IUT), ability to place multiple microcoils without cumulative risk, and lack of collateral damage that can occur with liquid agents or thermal energy. Limitations that have been described include the potential that the microcirculation supplied by collaterals of the feeder vessels remains intact, accidental occlusion of nontarget vessels, coil migration, and vessel perforation [12, 57, 58, 59, 60]. Additionally, involvement of an interventional radiology (IR) team is recommended if available as the procedure has been described as shorter in duration and more straightforward with the direct input of interventional radiologists at the time of microcoil placement [58, 60].

Ultrasound‐guided interstitial laser ablation (ILA) and thermal ablation with radiofrequency and microwave energy have also been described in several case reports [21, 61, 62, 63]. Bhide et al. reported the first known successful treatment of a placental chorangioma using ILA in 2003 [61]. ILA involves the placement of a 17‐gauge needle under ultrasound guidance near the hilum of the tumor followed by the passage of a laser fiber through the needle to allow the tip of the laser fiber to be adjacent to the feeder vessel. Once in position, the feeder vessel is then coagulated using laser energy. Benefits of this technique include a low risk of vessel rupture since the laser fiber is placed in the vicinity of the target vessel(s) rather than within, and the energy is transmitted through the placental tissue. Limitations include the risk of circumferential spread of the laser energy from the fiber tip, potentially damaging the umbilical cord and its vessels. Also, ILA does not allow visualization of vessels and may be insufficient for definitive treatment in severe cases [63].

Radiofrequency ablation (RFA) is a minimally invasive treatment that has been demonstrated as a reliable method for creating thermally induced coagulation necrosis in several different settings. Yulia et al. described a successful treatment of placental chorangioma using RFA in 2019. RFA employs targeted high‐energy electrical frequencies that generate heat within the tissue to destroy the tumor cells. After a prolonged period of sustained tissue temperature, coagulation of cellular enzymes and nucleic acid strands occurs, which leads to permanent cellular destruction [64].

Benefits of RFA include a minimally invasive approach and the ability to treat deep vessels within the tumor, rather than just superficial feeding vessels. Similar to ILA, RFA incurs the risk of accidental ablation of fetal cord vessels and heat transmission to nearby structures, causing unintentional side effects, fetal bleeding, exsanguination, and even fetal death [59].

A third thermal energy technique that may be used for the treatment of chorangioma is microwave ablation (MWA). Similar to RFA, MWA is a percutaneous, ultrasound‐guided procedure that achieves tumor devascularization. Reported advantages of MWA over RFA are a faster procedure, larger ablation zones, higher temperatures, less heat sink effect, and no need for grounding pads. In a retrospective comparison of RFA and MWA for the treatment of complicated monochorionic twins, both techniques achieved the primary objective of selective feticide with comparable survival rates and neurodevelopmental outcomes; however, MWA was associated with a lower risk of preterm birth [65]. Further research regarding MWA use for the treatment of chorangioma is warranted.

#### 7.2.2 Endoscopic treatments

Several techniques aimed at devascularization of the chorangioma with the aid of fetoscopy have been described including bipolar coagulation, vascular occlusion with placement of vascular clips or suture, and laser photocoagulation [1, 16, 21, 41, 43, 46, 66, 67]. In general, fetoscopic guidance allows for direct visualization of the target vessels and photocoagulation under fetoscopic control. Bouchghoul et al. examined 14 cases reported in the literature that were treated with fetoscopic laser photocoagulation between 2002 and 2015. In this review, the authors reported a live birth rate of 71% (10/14) and term delivery in 70% (7/10) of cases [68]. Although these reports are favorable, it should be noted that only one fetus in this cohort had developed hydrops prior to the procedure. As emphasized by Liu et al., interpretation of survival statistics must consider the predefined indications for surgery—that is, the degree of fetal compromise before intervention [29].

Although fetoscopic techniques may be a reasonably safe option in experienced hands, several limitations are important to highlight. First, the placement of a fetoscope requires a larger diameter cannula (typically 12 Fr) compared to ultrasound‐guided needle‐based techniques, which subsequently increases the risk of iatrogenic rupture of membranes. Other limitations include decreased visibility and technical feasibility in the setting of an anterior placenta, risk of thermal spread or collateral damage (especially if the targeted vessels are in close proximity to the umbilical cord insertion site), and increased risk of vessel rupture and hemorrhage—especially in the setting of large caliber and deep‐feeding vessels [16, 21, 29, 41, 46, 64, 69, 70].

In conclusion, although fetal therapy seems to have a role in the management of giant chorangioma and suspected fetal compromise, evidence regarding the optimal treatment strategy is lacking. Many authors have emphasized that the choice of surgical technique should take into account several factors, including gestational age, tumor size, vascularity, placental position, and the location of the chorangioma relative to the umbilical cord insertion. Table 4 presents a comparison of outcomes across different types of definitive interventions, including laser therapy, embolization, and radiofrequency ablation.

**TABLE 4:** Characteristics and outcomes of patients with large placental chorangiomas, stratified by intervention.

								Perinatal survival				
Study	Number of patients	Mean tumor diameter, cm	Mean GA (weeks) at diagnosis	Mean GA (weeks) at intervention	Mean GA (weeks) at delivery	IUT	IUFD	RFA	Laser (FLP or ILA)	US‐guided embolization/coils	Bipolar	Surgical clip
Agarwal 2023	9	8.02	22.9	24.5	37.1	5	4	1	8	0	0	0
Lim 2015	5	9	23.6	24.0	35.4	0	2	1	2	0	2^a^	1
Sepulveda 2009	3	7	—	27.0	32.5	1	1	0	3	0	0	0
Emery 2018	2	10.2	24.2	NR	37.0	2	1	0	0	2	0	0
Saeed 2023	2	7.5	25.8	NR	30.1	2	1	1	0	1	0	0
Bouchghoul 2021	2	5.3	22.7	23.0	36.6	0	0	0	2	0	0	0
Sanz Cortes 2022	2	7.35	23.5	24.0	37.0	2	0	0	0	2	0	0

Abbreviations: FLP, fetoscopic laser photocoagulation; GA, gestational age; ILA, interstitial laser ablation; IUFD, intrauterine fetal demise; IUT, intrauterine transfusion; NR, not reported; RFA, radiofrequency ablation.

^a^ One patient underwent Biopolar + RFA.

## 8 DELIVERY

The decision regarding the time and mode of delivery should consider the presence of serious maternal or fetal complications throughout pregnancy. In general, delivery at term is recommended unless fetal and/or maternal complications exist. While cesarean delivery may be indicated in some cases, patients with simple placental chorangioma may still attempt vaginal delivery, even in the presence of a giant chorangioma. Delivery should be planned with input from both maternal‐fetal medicine and neonatology. Upon delivery, the placenta should be assessed, with histopathologic evaluation by a pathologist recommended.

## 9 NEONATAL CARE

Given the significant cardiac demands placed on the fetus in the setting of a large chorangioma, it is imperative to relay information about the antenatal course to the neonatal team to ensure that they are prepared to manage a potentially critically ill neonate. Blood products should be on hand to manage anticipated neonatal anemia and thrombocytopenia.

In addition, several case reports have described a correlation between the presence of placental chorangiomas and the incidence of neonatal hemangiomas, which are the most common tumors of childhood [71, 72]. Although a more recent case‐control study (2017) did not demonstrate a statistically significant difference in the incidence of infantile hemangiomas between cases of chorangioma and controls, neonatologists should be informed of the antenatal course to guide the need for postnatal evaluation [73].

## 10 FUTURE DIRECTIONS

A multicenter, prospective observational study is currently underway through the North American Fetal Therapy Network (www.naftnet.org). Over time, this effort aims to better define the natural history of chorangiomas, guide evidence‐based surveillance strategies, refine selection criteria for intervention, and establish best practices for management.

### 10.1 Limitations

This article is a narrative review that synthesizes published literature and expert opinion to provide a comprehensive overview of current knowledge on placental chorangioma. As such, it does not follow a systematic search strategy, and potential publication bias, predominance of case reports, and heterogeneity of available evidence should be acknowledged when interpreting the conclusions.

## CONFLICT OF INTEREST STATEMENT

The authors declare no conflicts of interest.

## FUNDING INFORMATION

The authors received no specific funding for this work other than internal departmental resource.

## Data Availability

Data sharing is not applicable to this article as no datasets were generated or analyzed during the current study.
